# Real-Time MRI-Guided Catheter Tracking Using Hyperpolarized Silicon Particles

**DOI:** 10.1038/srep12842

**Published:** 2015-08-04

**Authors:** Nicholas Whiting, Jingzhe Hu, Jay V. Shah, Maja C. Cassidy, Erik Cressman, Niki Zacharias Millward, David G. Menter, Charles M. Marcus, Pratip K. Bhattacharya

**Affiliations:** 1 Department of Cancer Systems Imaging, The University of Texas MD Anderson Cancer Center, Houston, TX 77030; 2 Department of Bioengineering, Rice University, Houston, TX 77030; 3 Department of Biomedical Engineering, The University of Texas at Austin, Austin, TX 78712; 4 Kavli Institute of NanoScience, Delft University of Technology, Delft, Netherlands; 5 Department of Interventional Radiology, The University of Texas MD Anderson Cancer Center, Houston TX 77030; 6 Department of Gastrointestinal Medical Oncology, The University of Texas MD Anderson Cancer Center, Houston TX, 77030; 7 Niels Bohr Institute, University of Copenhagen, Denmark

## Abstract

Visualizing the movement of angiocatheters during endovascular interventions is typically accomplished using x-ray fluoroscopy. There are many potential advantages to developing magnetic resonance imaging-based approaches that will allow three-dimensional imaging of the tissue/vasculature interface while monitoring other physiologically-relevant criteria, without exposing the patient or clinician team to ionizing radiation. Here we introduce a proof-of-concept development of a magnetic resonance imaging-guided catheter tracking method that utilizes hyperpolarized silicon particles. The increased signal of the silicon particles is generated via low-temperature, solid-state dynamic nuclear polarization, and the particles retain their enhanced signal for ≥40 minutes—allowing imaging experiments over extended time durations. The particles are affixed to the tip of standard medical-grade catheters and are used to track passage under set distal and temporal points in phantoms and live mouse models. With continued development, this method has the potential to supplement x-ray fluoroscopy and other MRI-guided catheter tracking methods as a zero-background, positive contrast agent that does not require ionizing radiation.

In the United States, heart disease has been the leading cause of death for nearly a century^1^, with recent annual death tolls of approximately 600,000 people^2^ and direct and indirect costs exceeding $100 billion^3^ per annum. Cardiovascular diagnostic and interventional methodologies require the use of endovascular catheterization for procedures such as angiography, angioplasty, ablation, stent placement, and valve repair. Furthermore, catheters are also frequently used in risk stratification of chemotherapy-induced cardiotoxicity^4^ and embolization therapy of cancer patients^5^. Critical tracking of these catheters is typically accomplished by monitoring a radiopaque filler material embedded into the polymer walls of catheters using x-ray fluoroscopy^6^; this cardiovascular guidance approach allows for real-time feedback, high spatiotemporal resolution, and the ability to distinguish the position of the catheter relative to anatomical structures. However, x-ray fluoroscopy-guided catheter tracking suffers from limitations in soft tissue contrast, as well as difficulty in three-dimensional navigation^6^. To some extent, this is addressed using cone-beam CT image reconstruction, but with the added costs of increased radiation exposure and decreased soft tissue contrast. In the clinic, this technique typically requires refresh rates of 1–10 frames per second (FPS); these refresh rates, combined with procedure-related activities, can expose both the patient (direct exposure in the short term) and attending physician and team (scatter exposure over the long term) to ionizing radiation in a relatively short period of time (minutes to tens of minutes). This can be especially problematic for pediatric patients^7^, who not only have a much longer anticipated lifetime but also a greater potential for multiple procedures. Additional health concerns in patients that are attributed to the iodinated contrast media include nephropathy^8^ and, less commonly, allergic reactions.

Magnetic resonance imaging (MRI)-guided catheter tracking is attractive due to its many potential benefits, including three-dimensional imaging of the interactions between soft tissues and the vasculature without using ionizing radiation. The use of MRI-based catheter guidance also allows clinicians to simultaneously monitor other physiologically-relevant criteria, including metabolism, temperature, blood flow velocity, and tissue perfusion^9^. To date, typical MRI-guided catheter guidance approaches fall into one of two categories: active or passive tracking. The former method involves monitoring the active signal of a miniature radiofrequency (rf) coil placed near the catheter tip^10^, while the latter may examine susceptibility differences between paramagnetic dysprosium oxide rings embedded into the catheter versus that of nearby tissue^11^. Other passive MR catheter tracking techniques include *T*_1_-weighted imaging of a catheter filled with gadolinium^12^, or non ^1^H-imaging of catheters filled with other contrast media (including ^19^F imaging of perfluorooctylbromide^13^ and ^13^C imaging of hyperpolarized (HP) ^13^C-labelled 2-hydroxyethylpropionate^14^). While these methods offer contrast between the otherwise MR-invisible catheter and patient anatomy, they also suffer from inherent drawbacks that limit their applicability in the clinic. For example, active catheter tracking methods require specialized catheters and dedicated rf circuitry/equipment, while posing the risk of localized tissue heating and steering problems due to the inflexibility of the catheter tip^10^. While passive susceptibility tracking is a relatively simple process by comparison to active imaging, it usually provides negative contrast that is vulnerable to distortion artefacts^11^ and also requires the use of a specialized catheter. *T*_1_-weighted imaging of gadolinium-filled catheters requires competition with a significant ^1^H noise background and *T*_2_*-associated signal losses^12^. In the case of hyperpolarized ^13^C tracer alternatives, a continuous supply of the contrast agent is required because these tracers naturally depolarize within a timeframe of 60 seconds, an effect that is hastened by magnetization-depleting rf pulses during signal acquisition^14^ (a consequence that is true for all hyperpolarized media). Also, catheters that are filled with liquid MRI contrast agents (such as gadolinium or ^19^F and ^13^C tracers) cannot easily be used for simultaneously injecting other liquids into the body^13^ without employing multi-lumen catheters, thereby limiting their clinical use for further diagnostic and/or interventional procedures.

A method of hyperpolarizing silicon micro- and nanoparticles has been recently demonstrated^15^,^16^ to increase ^29^Si MR signals by up to 3–5 orders of magnitude via enhanced nuclear spin alignment, while retaining this improved signal for tens of minutes. Hyperpolarization of the ^29^Si nuclear spins is generated by solid-state dynamic nuclear polarization (DNP), which uses low temperatures and high magnetic fields to spin-polarize an electron bath to near unity; this spin polarization is then transferred to nearby nuclear spins through microwave-mediated dipolar interactions^17^. DNP of solid (dry) silicon particles takes advantage of naturally-occurring electronic defects on the particle surfaces and obviates the need for additional radicals to generate the necessary free electrons^18^. The resulting increase in ^29^Si nuclear spin polarization is relatively long-lasting (*T*_1 _~ 40 minutes)^15^ compared to other hyperpolarized modalities (e.g., HP ^13^C tracers)^19^, and is not affected by the *in vivo* environment. Silicon micro- and nanoparticles are non-toxic, non-radioactive, and have been investigated for biomedical applications due to their favorable biocompatibility and biodegradability^20^.

Here, we use solid-state hyperpolarized silicon particles as a proof-of-concept for MRI-based catheter guidance in both phantoms and *in vivo*. We demonstrate catheter tracking both over long time durations (40 minutes) and in real time (refresh rate of 6.25 FPS), as well as two-dimensional and three-dimensional catheter guidance visualization. This method of passive catheter tracking provides background-free positive contrast using a standard medical-grade catheter and does not require the catheter to be filled with a liquid tracer. The biocompatible silicon particles are commercially available and would contribute minimally to the cost of the procedure (the work presented here required ~3¢ of silicon particles), and are hyperpolarized using a well-characterized^21^ modality that has recently been made available for clinical studies of ^13^C-labeled metabolic tracers^22^. With further development, this approach could have a situational clinical role as a non-ionizing, zero-background, positive contrast imaging agent for real-time catheter guidance using MRI.

## Results

### Catheter tracking over long time durations

Silicon particles (average mean diameter ~ 2 μm) were packed into sample tubes and hyperpolarized in the solid state using a home-built DNP device. Following hyperpolarization, the particles were collected, quickly warmed to room temperature, and affixed to the tip of a medical grade catheter. For this study, two silicon samples were used: ~50 mg of particles loaded onto a 24 Fr urinary catheter (8 mm outer diameter, or ‘OD’), and ~6 mg of particles loaded onto a 5 Fr angiocatheter (1.67 mm OD). Additional experimental criteria are available in the Materials and Methods section, as well as the Supplementary Material.

**Figure 1:**
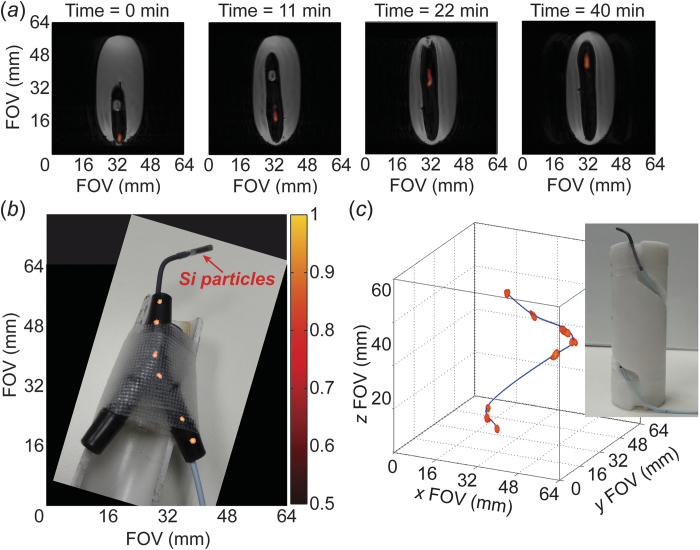
HP ^29^Si particle MRI-tracking in phantoms. (**a**) Transit of ~50 mg of silicon particles loaded into a 24 Fr urinary catheter moving ~4 cm through a gelatin phantom over the course of 40 minutes; co-registered ^29^Si/^1^H imaging shows the outline of the catheter in the void space left in the gelatin. (**b**) Angiocatheter (5 Fr) loaded with ~6 mg of silicon particles moving through Y-shaped hollow plastic phantom to simulate branching of vasculature; picture of catheter and phantom superimposed with a composite of ^29^Si MRI images. The sample tube containing silicon particles is push-fit onto the tip of the angiocatheter. (**c**) Angiocatheter tracking three-dimensional passage around a spiral phantom (*picture inset*). Absolute ^29^Si signal intensities (colored scale, arbitrary units) are consistent for (**a**)–(**c**); greyscale denotes ^1^H intensities. Pertinent imaging parameters, as well as Supplemental Video S1 (showing a rotating view of Fig. 1c), are included in the Supplementary Materials.

As an initial proof-of-concept, Fig. 1a shows positive contrast ^29^Si images (co-registered with ^1^H imaging) of the urinary catheter transiting ~4 cm through a gelatin phantom over the course of 40 minutes; this short distance is necessitated by the use of a ^29^Si/^1^H dual-tuned MRI coil that was designed for *in vivo* mouse studies (active region of coil only 52 mm in z-axis). The extended time scale over which the particles retain their increased magnetization is consistent with previous silicon micro- and nanoparticle studies^15^,^23^, and is far greater than what is typically expected from other hyperpolarized species (e.g., *T*_1_ of HP ^13^C-labelled tracers is typically ≤1 minute)^24^. The ability to acquire images over this time duration supports this method’s future development for potential utility in the clinic.

### Multi-dimensional catheter tracking

Following the initial catheter tracking demonstration using a large urinary catheter, we progressed to monitoring a medical-grade angiocatheter using roughly an order of magnitude fewer particles (corresponding to ~12% of the previously available magnetization). This 5 Fr catheter was tracked in two dimensions at distinct points over the course of ~4 cm and 28 minutes as it transited through a plastic Y-shaped hollow phantom to simulate guidance through the branching of the vasculature (specifically, for typical retrograde common femoral artery access with the catheter tip positioned above the level of the simulated aortic bifurcation; Fig. 1b). Further visualization of angiocatheter maneuverability includes three-dimensional tracking through a spiral-shaped phantom (Fig. 1c; Supplemental Video S1).

### *In vivo* catheter tracking

**Figure 2:**
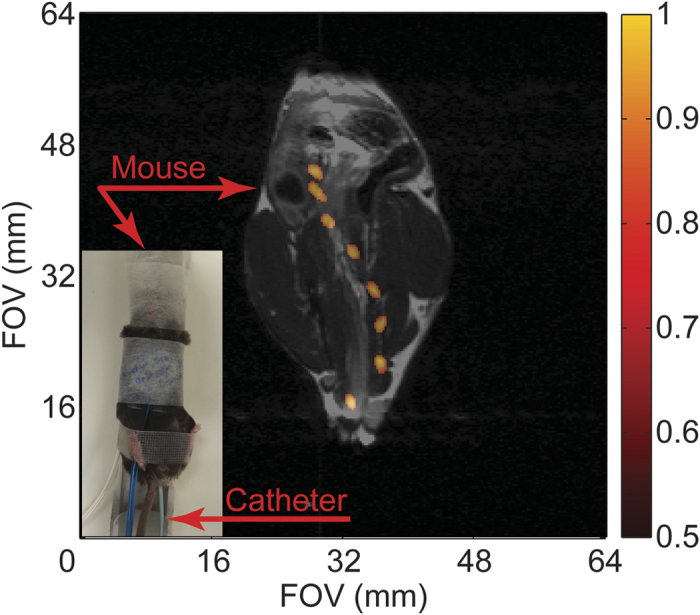
HP ^29^Si particle MRI-tracking *in vivo*. Composite of ^29^Si images (co-registered with single ^1^H anatomical scan) showing transit of angiocatheter loaded with silicon particles through the large intestines of a live normal mouse (*picture inset*) over the course of 4 min. Absolute ^29^Si signal intensities are denoted in arbitrary units on the colored scale; greyscale denotes ^1^H intensities. Pertinent imaging parameters, as well as Supplemental Video S2 (showing a time-lapse video of the catheter tracking in Fig. 2), are included in the Supplementary Materials.

**Figure 3:**
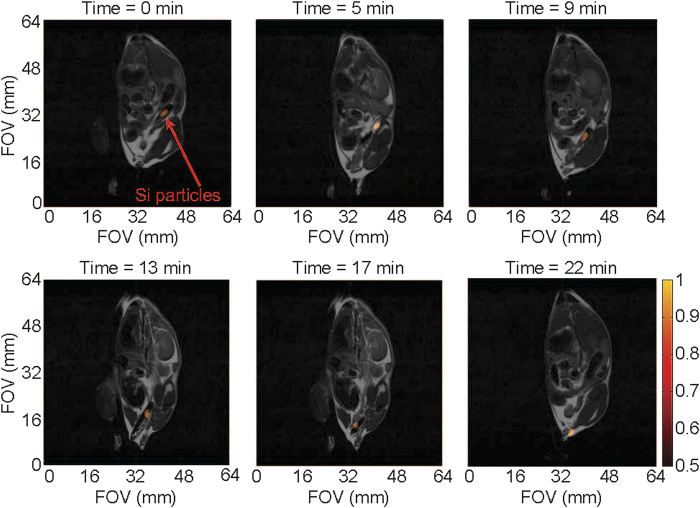
Co-registered ^29^Si/^1^H MRI-tracking *in vivo*. Transit of angiocatheter through the large intestines of a live normal mouse using alternating ^29^Si/^1^H scans, showing changes in mouse anatomy with movement of the catheter. Absolute ^29^Si signal intensities are denoted in arbitrary units on the colored scale; greyscale denotes ^1^H intensities. Pertinent imaging parameters, as well as Supplemental Video S3 (showing a time-lapse video of the catheter tracking in Fig. 3), are included in the Supplementary Materials.

Given that the 5 Fr angiocatheter is similar in diameter to commercially available endoscopes used for mouse colonoscopies^25^ as well as being a common size for human endovascular use, initial *in vivo* studies were carried out using the large intestine of a live mouse as a surrogate for the human vasculature. The 5 Fr angiocatheter, loaded with silicon particles, was inserted into the rectum of a normal mouse and a series of ^29^Si imaging acquisitions was executed at discrete intervals while the catheter transited through the intestinal tract (Fig. 2; Supplemental Video S2; Supplemental Fig. S1). Following this series, a single ^1^H image was taken for anatomical co-registration; because the ^1^H image was acquired following the catheter movement, there is a slight discrepancy in the overlaid images due to a catheter-induced shifting of the large intestines, along with potential peristaltic responses by the gut that are not present in the single ^1^H scan. Subsequent studies utilized an alternating ^29^Si/^1^H imaging protocol (Fig. 3; Supplemental Video S3) that shows the undulating of the intestines with the movement of the catheter. Regardless, the catheter is visualized moving in two dimensions ~3 cm through the intestinal tract of the mouse over the course of ~4 minutes (~2 cm in 22 min. for Fig. 3), demonstrating the first *in vivo* results using HP ^29^Si particles for catheter guidance.

### Real-time catheter tracking

**Figure 4:**
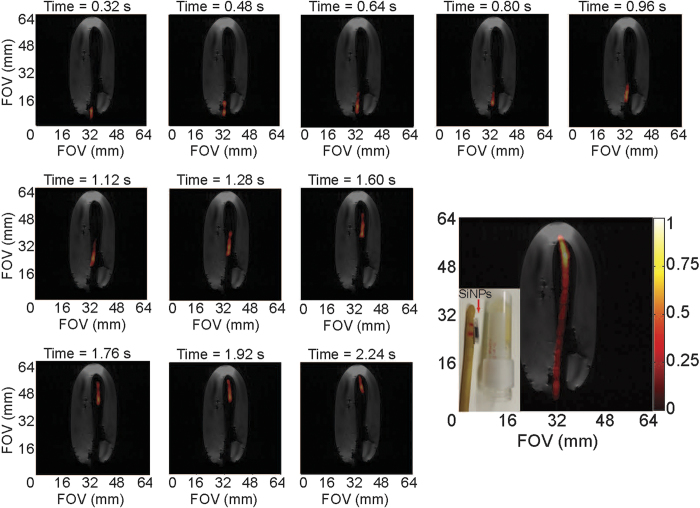
Real-time ^29^Si MRI catheter tracking. Individual scans showing movement of the large urinary catheter through a gelatin phantom at a frame rate of 6.25 FPS; bottom right figure shows composite of all twenty ^29^Si images (not all shown individually) over the course of 3.2 seconds. Co-registered with a single ^1^H scan (greyscale) after conclusion of ^29^Si images (colored scale). *Inset* picture shows silicon particles inside polarizing tube next to urinary catheter and gelatin phantom; during the experiment, the sample tube containing the silicon particles is placed inside the urinary catheter (utilizing the existing port near the catheter tip, *not shown*), where it rests between the two red horizontal lines drawn on the catheter. Pertinent imaging parameters, as well as Supplemental Video S4 (showing a real-time video of the catheter tracking in Fig. 4), are included in the Supplementary Materials.

Because continuous imaging is requisite for catheter tracking in the clinic, we demonstrated this technique using real-time ^29^Si imaging of the urinary catheter transiting through a gelatin phantom (Fig. 4; Supplemental Video S4). The co-registered images show the catheter moving ~5 cm over the course of 20 frames in ~3.2 seconds (only 11 of the 20 frames shown here), resulting in a frame rate of 6.25 FPS. These image refresh rates are comparable to those that are typically achieved using fluoroscopy-guided catheter tracking in the clinic^6^. Longer time durations of continuous imaging are also possible using the same allotment of hyperpolarized particles (as the experiment was successfully repeated immediately afterwards with the same sample; *not shown*).

## Discussion

In this proof-of-concept study, we have demonstrated the viability of passive catheter tracking using hyperpolarized ^29^Si MRI using both phantoms and mouse models, over long time durations and in real time, and in both two and three dimensions. This method provides radiation-free, background-free positive contrast over the course of >40 minutes using non-specialized catheters that are tagged with a biologically safe media. While current limitations in ^29^Si polarization level, MR hardware, and MR pulse sequences did not allow for real-time imaging (of several FPS) over the course of minutes, this advance will be critical to potential future clinical translation. With further development, co-registered ^1^H/^29^Si MRI may find a role in clinical catheter tracking because of its ability to image the tissue/vasculature interface, as well as track other physiologically-relevant criteria. Compared to other MRI-guided catheter imaging techniques, it is not susceptible to rf burns, negative contrast, distortion artefacts, or competitive background signals.

The experiments presented here are limited by a reduced field of view that is inherent to small animal imaging coils that are designed for mouse imaging (35 mm inner diameter, or ‘ID’; 52 mm homogenous rf region in z-axis); this is a result of the ^29^Si DNP polarizer being situated in a small animal imaging facility without any clinical scanners in close proximity. Combined with this is a current lack of human-scale MRI detection coils that are tuned to the ^29^Si resonance frequency. Another potential drawback is the non-renewable nature of hyperpolarized signal; which decays through both natural spin population redistribution over time, as well as through the application of magnetization-depleting rf pulses for image acquisition. The ^29^Si signal in these particles can last for tens of minutes, which is on the same scale as most endovascular catheterization procedures. Also, the depletion of available magnetization upon the administration of rf pulses was mitigated by using small tipping angle pulses to minimally perturb the ^29^Si spins. For early acquisitions, a Fast Low-Angle Shot (FLASH) sequence employed a ramped tipping angle to provide near-constant ^29^Si signal intensities with each acquisition; the final image of the longer time duration experiments ended with a 90° Rapid Acquisition with Refocused Echos (RARE) sequence to maximize the amount of signal left at the end of the study. Given the short *T*_2_* of these silicon particles (~600 ms), additional gains could be achieved by employing a zero echo time (ZTE) imaging sequence to improve the signal-to-noise ratio with even smaller excitation pulses; it should also be noted that ^29^Si MRI scans were completed with single scans (no averaging required) and use high reconstruction thresholding due to zero ^29^Si signal background and *a priori* knowledge of the expected image profile. Furthermore, the long *T*_2_ (1–2 s) of these could be taken advantage of in order to image more lines of k-space with fewer rf pulses, and future improvements to the imaging hardware (i.e., flexible phased-array coils) can also be used to maximize scanning time by mitigating the deleterious effects of image acquisition. With moderate improvements to the ^29^Si hyperpolarization level, MR hardware, and pulse sequences, the spatial resolution in this study (~1 mm) may become more competitive with the current clinical standard of x-ray fluoroscopy (~0.1 mm).

Silicon-based micro- and nanoparticles have received recent interest as targeted diagnostic and drug delivery vehicles, due to their biocompatibility, biodegradability, and simple surface chemistry that is amenable to drug loading and targeting^20^. Because of this, they are favorable for development as platform nanotechnologies, where multiple targeting agents and therapeutic drugs can be attached to the particles surfaces for multiplexed theranostic applications. For this study, we chose larger silicon microparticles because of their longer *T*_1_ compared to particles in the <100 nm range (*T*_1 _~ 10–15 min). The ability to hyperpolarize these particles makes them amenable to *in vivo* MR imaging^15^; since its gyromagnetic ratio is similar to those of ^13^C and ^15^N, the ^29^Si resonance frequency is typically within the tuning range of commercial (multinuclear) MRI systems. Increasing interest in clinical ^29^Si MRI may prompt the implementation of human-scale imaging coils that are resonant at the ^29^Si precession frequency; these coils may be able to improve on relative sensitivity (neglecting filling factor) using phased-array receiver configurations. Future studies will look to utilize clinical MRI scanners and torso ^29^Si imaging coils to expand the available field of view for catheter tracking.

The recent clinical demonstration of DNP of small ^13^C-metabolites^22^ in prostate cancer patients, along with ongoing clinical trials of silicon-based particles for drug delivery^26^, should help pave the way for rapid translation of hyperpolarized ^29^Si MRI to the clinic. Although current versions of commercially-available clinical DNP devices are not marketed for silicon hyperpolarization, there should be no technical reason why it would not be feasible with minor alterations; in the future, using these devices for both ^13^C metabolic studies and ^29^Si molecular and interventional imaging could help defray hospital costs for access to hyperpolarized media. Furthermore, because the effects of hyperpolarization are field-independent, this technique is amenable for MRI at lower *B*_0_, as well as in open-configuration scanners that are more conducive to interventional procedures. For this proof-of-concept work, the sample tube of hyperpolarized silicon particles was either push-fit onto the end of the angiocatheter, or placed inside the end of the urinary catheter; while we did not physically alter the catheter in any way, we recognize that improvements in silicon particle placement will be key to further development. To that end, future studies will attempt to coat the entirety of the catheter in hyperpolarized silicon particles to permit visualization of the full catheter length (allowing bends and/or kinks to be monitored) while allowing the lumen to be used to inject contrast media, collect specimens, and conduct interventional operations and therapies. With further development, enhanced ^29^Si MRI-guided catheter visualization may allow clinicians to perform concurrent diagnostic and interventional MRI studies without the need to shuttle patients from one imaging suite to another, decreasing patient residence time and increasing safety.

## Materials and Methods

### ^29^Si particles and catheters

Silicon particles (polycrystalline/amorphous; average mean diameter ~2 μm) were commercially sourced (CAS No. 7440-21-3) and used as received (99.9985% elemental purity; ^29^Si isotopic natural abundance of ~4.7%). The particles were packed into small Teflon tubes; one sample (used for phantom experiments) contained ~50 mg of particles packed into a 3 mm ID × 8 mm long tube and (following ^29^Si DNP) was placed inside the existing opening near the tip of the large urinary catheter (24 Fr; 8 mm OD; Rochester Medical Corp.), while the other sample (used for phantom and mouse experiments) consisted of ~6 mg of particles packed into a 1.4 mm ID × 4.5 mm long tube and (following DNP) was push-fit onto the tip of the angiocatheter (5 Fr; 1.67 mm OD; Cook Medical). For hyperpolarization, the sample tubes were push-fit onto the end of a garolite rod and inserted into the DNP device (the smaller sample was placed inside of a larger sample tube, which was then push-fit onto the end of the garolite rod).

### ^29^Si DNP

After insertion of the packed sample tubes into the home-build polarizer, DNP was performed at ~ 3.2 K and ~2.9 T. Polarization times typically ranged from 5 hours for the larger (50 mg) sample to 17 hours for the smaller (6 mg) sample; the deciding factor for polarization time was the ability to generate sufficient ^29^Si signal to complete the imaging study (these silicon particles typically reached steady-state hyperpolarization after ~15 hrs of DNP). The 100 mW microwave source was frequency-modulated from 80.83 to 80.90 GHz using a 20 kHz ramp modulation, and directed to the sample via waveguide and slot antenna. Quality control was monitored using an on-board miniature NMR spectrometer to sample ^29^Si polarization levels during DNP. The silicon particles can be quickly removed from the polarizer, warmed to room temperature, and affixed to the catheter tip without a significant loss in polarization; the low specific heat capacity (712 J/kg°C) and robust thermal conductivity (159 W/m°C) of silicon^27^ allow the sample to be warmed by hand while transporting to the MRI scanner (*T*_transport _< 1 minute). The measured hyperpolarized relaxation rate of the silicon particles was ~25 minutes at 7 T and room temperature.

### MRI experiments

All imaging experiments described here were performed in a 7 T horizontal-bore small animal scanner (Bruker Biospin), using Paravision software (v5.1; Bruker Biospin). A custom-made dual-tuned ^1^H/^29^Si litz coil (Doty Scientific) was used for co-registered imaging (35 mm ID; homogenous rf region ~52 mm along z-axis). A small sample of silicon oil (1.5 ml; CAS: 63148-62-9) was used for calibration purposes; typical ^29^Si nuclear spin polarization values ranged from 0.5–1.0%. ^29^Si imaging was performed using Fast Low Angle Shot (FLASH) and Rapid Acquisition with Refocused Echoes (RARE) sequences; ^1^H anatomical and phantom images used a RARE sequence in the coronal plane. Additional details of the imaging sequences and processing protocols are listed in the Supplementary Materials.

### Phantom experiments

Phantoms were positioned in the center of the homogenous rf region of the MRI coil, and the HP ^29^Si -tagged catheter was moved through the phantom during imaging acquisitions. Phantoms consisted of gelatin inside a 50 ml centrifuge tube (Figs 1a and 4), a 3-way plastic hose barb connector (Fig. 1b), and a spiral groove etched into the side of a 32 mm diameter × 98 mm long cylindrical stock of PTFE (Fig. 1c).

### Mouse handling

All animal studies were performed in accordance with animal use protocols that were approved by the UT MD Anderson Cancer Center “Institutional Animal Care and Use Committee” (IACUC). Wild type male APC^(+/+)^ mice with a BL6 background (DOB 12/25/2013; sourced from MD Anderson Cancer Center) were used in all studies; these non-genetically modified mice (tail genotyping) were produced in an APC^MIN^ breeding colony. These normal mice were anesthetized with 2% isoflurane (in 0.75 l/min oxygen) administered by an MR-compatible nose cone while the mouse was stationed on a custom cradle inside the MRI coil. The HP ^29^Si -tagged 5 Fr angiocatheter was inserted ~3 cm into the rectum of the live mouse; it was then slowly pulled out in discrete intervals corresponding to the given imaging sequence. For Fig. 2; a single ^1^H image was acquired after the series of ^29^Si images. For Fig. 3, alternating ^29^Si and ^1^H images were acquired. All mice survived the procedure with no evidence of ill effects.

## Additional Information

**How to cite this article**: Whiting, N. *et al.* Real-Time MRI-Guided Catheter Tracking Using Hyperpolarized Silicon Particles. *Sci. Rep.* **5**, 12842; doi: 10.1038/srep12842 (2015).

## Supplementary Material

Supplementary Information

Supplemental Video 1

Supplemental Video 2

Supplemental Video 3

Supplemental Video 4
