# Supplementary material for: Real-Time MRI-Guided Catheter Tracking Using Hyperpolarized Silicon Particles
Source: Sci Rep. 2015 Aug 4;5:12842. doi: 10.1038/srep12842 (PMC4523869; doi:10.1038/srep12842)
Supplement: Supplementary Information [file srep12842-s1.pdf]

Supplementary Material for:

Title: Real-Time MRI-Guided Catheter Tracking Using Hyperpolarized Silicon Particles

Authors: Nicholas Whiting[1†], Jingzhe Hu[1,2†], Jay V. Shah[1,3], Maja C. Cassidy[4], Erik Cressman[5], Niki Zacharias Millward[1], David G. Menter[6], Charles M. Marcus[7], Pratip K. Bhattacharya[1]*

Affiliations:
[1]Department of Cancer Systems Imaging, The University of Texas MD Anderson Cancer Center, Houston, TX 77030
[2]Department of Bioengineering, Rice University, Houston, TX 77030
[3]Department of Biomedical Engineering, The University of Texas at Austin, Austin, TX 78712
[4]Kavli Institute of NanoScience, Delft University of Technology, Delft, Netherlands
[5]Department of Interventional Radiology, The University of Texas MD Anderson Cancer Center, Houston TX 77030
[6]Department of Gastrointestinal Medical Oncology, The University of Texas MD Anderson Cancer Center, Houston TX, 77030
[7]Niels Bohr Institute, University of Copenhagen, Denmark

*[)]To whom correspondence should be addressed. Electronic mail:

pkbhattacharya@mdanderson.org; fax: 713-563-4894

[†)]These authors contributed equally to this work.

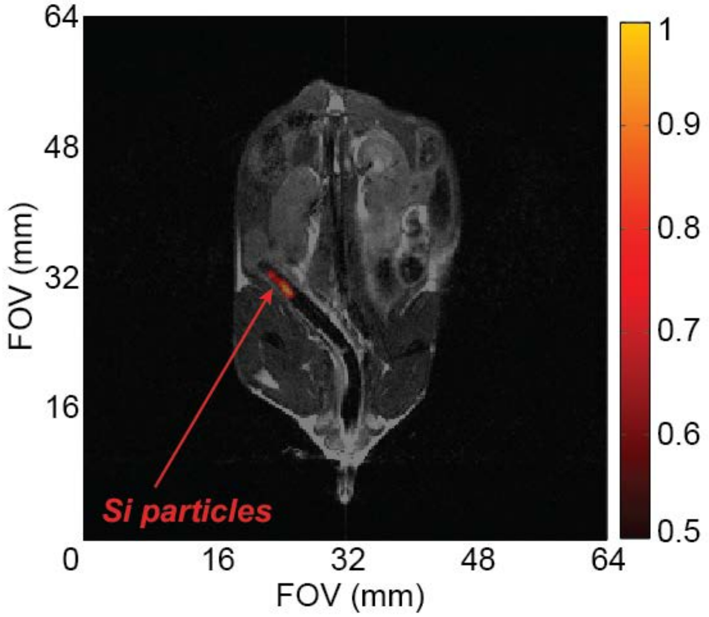

Supplemental Fig. S1: High resolution HP ^{29}Si particle MRI-tracking *in vivo*. ^{29}Si image (co-registered with single ^{1}H anatomical scan) showing a high resolution (1 mm x 1 mm) ^{29}Si image of an angiocatheter loaded with silicon particles inside the large intestines of a live normal mouse. Single 90° ^{29}Si RARE image (TR/TE: 500 ms/2.184 ms; RARE factor of 64). Absolute ^{29}Si signal intensities are denoted in arbitrary units on the colored scale; greyscale denotes ^{1}H intensities. See *inset* of Fig. 2 for example photograph of mouse.

Imaging methods and processing

Fig. 1a shows four images using ^{29}Si FLASH (α = 8°, 8°, 12°) and RARE (α = 90°) imaging sequences (at t = 40 min), respectively. The FLASH images used TR/TE values of 2 ms/0.7 ms, while the RARE image used TR/TE values of 60 ms/1.841 ms; all ^{29}Si images were acquired with a 32 x 32 matrix size, FOV = 64 x 64 mm, and resolution of 2 x 2 mm. The ^{29}Si images are co-registered with a ^{1}H coronal RARE scan (α = 90°), TR/TE: 1800 ms/9.6 ms with a RARE factor of 8; 256 x 256 matrix size (0.25 x 0.25 mm resolution). **Fig. 1b** was achieved with a series of ^{29}Si FLASH sequences (α = 8-22°, 32x32) at discrete time intervals, with the last timepoint (28 minutes) corresponding to a 90° ^{29}Si RARE sequence. The picture of the catheter

and phantom was scaled and overlaid in Adobe Illustrator. **Fig. 1c** used a series of ^{29}Si FLASH sequences (3D 32x32x16) with α = 3-6°, TR/TE: 4 ms/0.662 ms, and resolution of 2 x 2 x 4 mm.

Figure 2 shows a composite of eight ^{29}Si FLASH images (α = 6-11°, 32x32), TR/TE: 4 ms/0.692 ms, 2 x 2 mm pixel size. Co-registered with an average of three ^{1}H coronal scans (RARE; α = 90°) taken after the conclusion of the ^{29}Si imaging protocol, TR/TE: 60 ms/1.841 ms, 2 x 2 mm pixel size.

Figure 3 shows a series of six ^{29}Si FLASH images (α = 6-10°, 32x32), TR/TE: 4 ms/0.692 ms, 2 x 2 mm pixel size. Co-registered with an average of three ^{1}H anatomical coronal scans (RARE; α = 90°), TR/TE: 1800 ms/9.6 ms with a RARE factor of 8, 0.25 x 0.25 mm pixel size, taken immediately after each ^{29}Si acquisition to monitor catheter-induced anatomical movement.

For Figures 1-3, the initial tipping angle was chosen to provide an adequate amount of signal for observation while minimally perturbing the available magnetization. The tipping angle was ramped for subsequent acquisitions to maintain a near-steady signal intensity. The final acquisition used the remaining magnetization with a hard 90° pulse.

Figure 4 used a sequential series of ^{29}Si FLASH sequences (repetition time = 5 ms, number of repetitions = 20, 32x32) with α = 4°, TR/TE: 5 ms/ 0.692 ms, resolution of 2 x 2 x 2 mm. This series of images took place over 3.2 seconds. Co-registered with a ^{1}H coronal RARE scan (α = 90°)

All data was processed in Matlab using the following procedure:

a) perform ^{29}Si image processing routine:

1. Zero-filling the original k-space data (2D dataset 32x32, 3D dataset 32x32x8) to 256x256 (x256) before Fourier transformation
2. Import images reconstructed by ParaVision into MatlabNormalize the image to [0,1]
3. Normalize the image to [0,1]
4. Apply a threshold to the image according to Supplemental Table1. We employed a relatively high threshold due to *a priori* knowledge that the hyperpolarized ^{29}Si signals emanate from one concentrated source.

b) perform ^{1}H image processing routine:

1. Import images reconstructed by ParaVision (default settings) into Matlab

2. Identify relevant slices and increase the contrast of ^{1}H images by saturating the top and bottom 1%.

c) overlay the ^{29}Si and ^{1}H image with the "Image Processing Toolbox" in Matlab

Supplemental Table1: Threshold used for ^{29}Si image processing

Figure	Description	Threshold
Fig. 1a	urinary catheter (large sample)	0.5
Fig. 1b	Y-phantom (small sample)	0.85
Fig. 1c	3-D spiral phantom (small sample)<br>Supplemental video 1	0.80
Fig. 2	*in vivo* (small sample)<br>Supplemental video 2	0.75
Fig. 3	*in vivo*, alternating ^{1}H, ^{29}Si images(small sample)<br>Supplemental video 3	0.75
Fig. 4	real time urinary catheter (large sample)<br>Supplemental video 4	0.5
SFig. 1	high resolution HP ^{29}Si particle MRI-tracking *in vivo*	0.5

Supplemental Video S1: Rotating view of spiral phantom (for Fig. 1c).

Supplemental Video S2: Time lapse video of *in vivo* ^{29}Si catheter tracking (for Fig. 2).

Supplemental Video S3: Time lapse video of co-registered *in vivo* ^{29}Si catheter tracking (for Fig. 3).

Supplemental Video S4: Real-time imaging of catheter transit in gelatin phantom (for Fig. 4).
